# Home High-Flow Therapy in Patients with Chronic Respiratory Diseases: Physiological Rationale and Clinical Results

**DOI:** 10.3390/jcm12072663

**Published:** 2023-04-03

**Authors:** Maria Laura Vega Pittao, Gioacchino Schifino, Lara Pisani, Stefano Nava

**Affiliations:** 1 Alma Mater Studiorum, Department of Medical and Surgical Sciences (DIMEC), University of Bologna, 40138 Bologna, Italy; 2 Respiratory and Critical Care Unit, IRCCS Azienda Ospedaliero Universitaria di Bologna, 40138 Bologna, Italy

**Keywords:** high-flow nasal cannula, HFNC, high-flow oxygen therapy, HFOT, NIRS, non-invasive respiratory supports, chronic respiratory disease, COPD

## Abstract

High-flow therapy (HFT) is the administration of gas flows above 15 L/min. It is a non-invasive respiratory support that delivers heated (up to 38 °C), humidified (100% Relative Humidity, RH; 44 mg H_2_O/L Absolute Humidity, AH), oxygen-enriched air when necessary, through a nasal cannula or a tracheostomy interface. Over the last few years, the use of HFT in critically ill hypoxemic adults has increased. Although the clinical benefit of home high-flow therapy (HHFT) remains unclear, some research findings would support the use of HHFT in chronic respiratory diseases. The aim of this review is to describe the HFT physiological principles and summarize the published clinical findings. Finally, we will discuss the differences between hospital and home implementation, as well as the various devices available for HHFT application.

## 1. Introduction

High-flow therapy (HFT) is usually defined as the administration of gas flows above 15 L/min. It is a non-invasive respiratory support that delivers heated (up to 38 °C), humidified (100% Relative Humidity, RH; >30 mg H_2_O/L Absolute Humidity, AH) [1,2], oxygen-enriched air when necessary, through a nasal cannula or a tracheostomy interface, and is typically administered in the 30–50 L/min range in clinical practice.

HFT was first introduced in the pediatric setting in the 1990s to treat apnea of prematurity as an alternative to nasal Continuous Positive Airway Pressure (CPAP), due to the distending pressure generated by nasal cannula flow in neonatal patients [3,4]. Additionally, in the 1990s, the first study showing that HFT improved exercise tolerance compared to low-flow oxygen in patients affected by chronic obstructive pulmonary disease (COPD) was published [5]. Years later, HFT gained popularity as an alternative method of respiratory support for critically ill hypoxemic patients. Nevertheless, the first systematic review published in 2010 included only eight articles, all of which were abstracts or poster presentations from scientific meetings, resulting in poor data quality for analysis [6]. Over the last few years, the use of HFT in critically ill hypoxemic adults has increased. The most recent systematic reviews and meta-analyses [7,8] have shown that, although HFT does not reduce mortality in patients with acute hypoxemic respiratory failure, it may reduce the need for intubation when compared to standard oxygen therapy (SOT), and that it is not inferior to NIV in terms of reducing the incidence of reintubation. High flow is now well studied, mostly in intensive care for hypoxemic patients. In other settings, however, research is ongoing. The clinical benefit of home high-flow therapy (HHFT) remains unclear. Nevertheless, some research findings would support the use of HHFT in chronic respiratory diseases because of its ease of implementation and patients’ perceived comfort. The purpose of this review is to describe the physiological principles of HFT and summarize the published studies (Table 1). Finally, we will discuss the differences between hospital and home implementation, as well as the various devices available for HHFT application. 

## 2. The Potential Mechanisms by Which HHFT May Offer Advantages to Stable Hypoxemic and/or Hypercapnic Patients

Mechanisms by which HHFT could enhance the breathing pattern in chronic respiratory diseases are [21,22]:

A—improved lung mucociliary clearance and decreased inspiratory resistance by providing heated and humidified gas;
B—washout of anatomic dead space;
C—mild distending pressure; and
D—increased alveolar PO_2._

### 2.1. A—Improvement in Lung Mucociliary Clearance and Attenuation of Inspiratory Resistance Provided by Heated and Humidified Gas

In normal conditions, as air moves from the nasopharynx to the carina (the isothermal saturation boundary), it becomes fully saturated with water vapor (AH = 44 mg/L and RH = 100%). These conditions are optimal for maintaining the normal functioning of the epithelial cells and the ciliary function. Studies have shown that deficient airway surface hydration may play a critical role in the pathogenesis of airway inflammation with chronic airway mucus obstruction [23,24]. Mall et al. [25] studied the natural progression of lung disease caused by airway surface dehydration in mice. They demonstrated that airway surface dehydration is sufficient to initiate persistent neutrophilic inflammation with chronic airway mucus obstruction and to cause transient eosinophilic airway inflammation and emphysema. This may suggest that deficient hydration may trigger the mechanisms leading to chronic pulmonary diseases of different etiologies. HFT provides heated humidified gas to enhance lung mucociliary clearance, decreasing the risk of atelectasis [24]. Kilgour et al. tested whether reducing the air temperature would affect mucus transport velocity and ciliary beat frequency. They conclude that delivering inspired gas at 30 °C or even 34 °C with 100% RH may not be enough to prevent epithelial damage in animal models after 6 h of exposure. Furthermore, when using FiO_2_ above 21%, the air temperature and ciliary beat may fall even further. Other problems associated with under-humidification are discomfort [26,27] and bronchoconstriction [28,29]. In addition, when inspiratory gas is drawn across the nose, retraction of the nasopharyngeal boundaries increases inspiratory resistance significantly [30]. Moreover, inhalation of cold and dry air activates specific receptors and osmoreceptors in the nasal mucosa, causing bronchoconstriction in both healthy individuals and COPD patients [28,31]. These features of heated, humidified HHFT may be crucial for chronically hypersecretory patients who require airway clearance optimization or for patients suffering from bronchial hyperreactivity. A classical example of this condition is bronchiectasis. It is a chronic respiratory disease defined as an abnormal and permanent dilatation of the bronchi, abnormal mucus production, and frequent respiratory exacerbations leading to poor quality of life, lung function deterioration, and chronic respiratory failure. Patients with a persistent productive cough or difficulties expectorating sputum should be encouraged to improve airway clearance [32]. Rea et al. demonstrated that HFT decreased exacerbation frequency and exacerbation days and increased time to the first exacerbation in a cohort of 108 stable patients diagnosed with COPD or bronchiectasis [9]. A post-hoc analysis regarding only patients with bronchiectasis (45 patients; 41.7%) revealed that the exacerbation rate was significantly reduced (2.39 vs. 3.48 exacerbations per patient per year; rate ratio 0.69; 95% CI 0.49–0.97; *p* = 0.03), and quality of life improved in the HHFT group, suggesting that HHFT is a potential treatment option for patients with bronchiectasis. Hasani et al. evaluated 10 bronchiectasic patients with HHFT (flow rate 20–25 L/m, temperature 37 °C); they proved, using radiomarked aerosolized particles at baseline and after 7 days of treatment, that warm air humidification treatment can increase mucociliary clearance [10]. In hospitalized patients with a diagnosis of acute exacerbation of COPD and concurrent bronchiectasis, HFT appeared to be beneficial in improving gas exchange and reducing respiratory rate and dyspnea [33].

Similarly, HFT delivered via a tracheostomy interface (tHFT) appears to improve the ciliary beat frequency and reduce the number of suctioning procedures required following a tracheostomy [34]. Dolidon et al. reported the characteristics of 71 patients discharged with HHFT in a retrospective study. In 39% of patients, HFT was delivered through a tracheostomy cannula or a tracheal mask (tHFT). The mean air flow and FIO_2_ delivered in this subgroup were 32.4 ± 12 L/m and 28.5 ± 9.9%, respectively. tHFT was mainly prescribed in order to improve mucociliary clearance and reduce the number of exacerbations. Following the start of tHFT, there was a reduction in the number of admissions due to low respiratory tract infection in this cohort [35].

### 2.2. B—Dead Space Washout

In a normally functioning lung, alveolar ventilation is near 70%. This “effective ventilation”, which participates in gas exchange, can be significantly reduced in various disease states. “Washing out” the expired air in the upper airways and decreasing CO_2_ rebreathing improves ventilation efficiency, resulting in alveolar ventilation with a higher proportion of minute ventilation. It was demonstrated that the CO_2_ clearance rate from the anatomical dead space is linearly related to the flow rate. A study using upper airway models showed that CO_2_ clearance was greater when the flow was increased from 15 to 30 L/min, rather than from 30 to 45 L/min [36]. In another study with healthy volunteers and tracheotomized patients, the investigators found a link between a decrease in CO_2_ rebreathing (by 1 to 3 mL per breath) and a similar increase in inspired O_2_, corresponding to a reduction in dead space by 20–60 mL after increasing the flow rate from 15 to 45 L/min [37]. A study of ten healthy volunteers found that dead space washout is present up to high flows of 40 L/m, identifying no further increase in washout at higher flows (60 L/m), assuming a “plateau effect” when flows are set above 40 L/m [38]. The existing literature demonstrates adequate physiological rationale to proceed with trialing these devices in the long-term management of COPD. For example, in a randomized crossover trial comparing HFT plus long-term oxygen therapy with SOT in patients with stable hypercapnic COPD, 6 weeks of treatment with HFT improved health-related quality of life and decreased the arterial partial pressure of carbon dioxide by at least 10% (adjusted treatment effect, −4.1 mm Hg; 95% confidence interval, −6.5 to −1.7 mm Hg), pH (adjusted treatment effect, +0.02; 95% confidence interval, 0.01 to 0.02), and median nocturnal transcutaneous carbon dioxide pressure (adjusted treatment effect, −5.1 mmHg; 95% confidence interval,−8.4 to −1.8 mm Hg) [11]. Similarly, compared to long-term oxygen treatment (LTOT), HHFT reduced the transcutaneous carbon dioxide (TcCO_2_) and respiratory rate in patients with stable oxygen-dependent COPD. The authors argued that the decrease in TcCO_2_ is related to the consistent rise in tidal volume, which is followed by dead space and CO_2_ washout [12]. Storgaard et al. carried out a post-hoc analysis from a previous randomized controlled trial comparing SOT vs. HHFT plus oxygen [13], in which they included 74 patients with concomitant persistent hypercapnic failure (>45 mmHg). After 12 months, there was a 1.3% decrease in PaCO_2_ in patients using HFT and a 7% increase in controls before HFT use on site (*p* = 0.003), concluding that HFNC stabilizes patients with COPD with persistent hypoxic and hypercapnic failures in terms of PaCO_2_, exacerbations, and number of hospitalizations, whereas those not receiving HFT worsened [14]. In line with these findings, Pisani et al. found that in COPD patients recovering from an episode of AHRF who had attained a normal pH, using HFT was related to a statistically significant drop in PaCO_2_ and respiratory rate. The subset of patients with a lower pH level had the best response. However, the authors of this study were able to establish that COPD patients with the overlap syndrome had a different response [15]. This evidence suggests that HHFT may be a viable alternative to SOT for stable hypercapnic patients with COPD.

Furthermore, HHFT has been compared to non-invasive mechanical ventilation (NIV) in 102 stable COPD patients during a 6-week crossover study. HFT may be a feasible alternative to NIV in terms of PaCO2 reduction and quality of life improvement in COPD patients who refuse NIV or are intolerant. Interestingly, this study showed that a significant subset of patients (around 15–20%) in both groups did not improve or even worsen their PaCO2. There were three patients whose PaCO2 levels increased by more than 5 mm Hg using both devices, five using only HHFT, and four using NIV [16]. This may suggest that when hypercapnia does not improve with one specific non-invasive support (either NIV or HHFT), a trial with the alternative method is mandatory. Indeed, HFT may be a viable alternative to SOT during NIV pauses since it may be more comfortable or result in better outcomes for dyspnea [39].

### 2.3. C—Provide a Mild Distending Pressure

This effect may optimize lung mechanics by improving lung compliance and gas exchange while maintaining alveolar patency. Distending pressure provided by HFT is dependent on the leak rate, which is determined by the nasopharyngeal anatomy as well as the relationship between nasal prong size and the nares of the nose and whether the mouth is open or closed [40]. Nonetheless, HFT distending pressure is unlikely to be above 2–4 cmH_2_O, and it does not likely deliver a clinically relevant level of positive pressure in terms of lung recruitment, as CPAP does [41,42]. Moreover, the mechanisms for increasing expiratory positive pressure differ between HFT and CPAP. HFT increases the expiratory resistance and may exert a jet-flow effect that creates a pressure gradient across the flow-restricted nose segment (zero at the nares and positive inside the nasal cavity), whereas CPAP increases pressure in the nares without creating a further pressure gradient and without affecting the expiratory resistance of the upper airway [43,44]. The increase in expiratory resistance leads to a longer expiratory phase, lowering the respiratory rate and minute ventilation [45]. Surprisingly, it may appear paradoxical that COPD patients could benefit from the mechanism of increasing expiratory resistance, but it is actually a similar effect to the pursed-lips breathing pattern adopted by these patients, which might be useful by keeping the airway open and improving exercise capacity [46]. Additionally, this mild positive airway pressure mentioned before may counterbalance intrinsic positive end-expiratory pressure (PEEP) in COPD patients and reduce the respiratory workload while also improving exercise tolerance [17].

When comparing HFT delivered through a nasal cannula (nHFT) vs. via a tracheal interface (tHFT), tHFT seems to generate even less distending pressure. In a bench trial, Thomas et al. demonstrated that tHFOT generates a potential PEEP of 0.3 cmH_2_O, 0.5 cmH_2_O, and 0.9 cmH_2_O when the flow is set to 40 L/m, 50 L/m, and 60 L/m, respectively [47]. In line with these findings, tHFOT produces lower tracheal mean and peak expiratory pressure when compared to nHFT [48]. Probably the main reason to justify the effect could be that tHFT is set up with a T-piece Mapleson system with one end connected to the tracheostomy tube and the other end left open to facilitate exhalation, resulting in an open-circuit system.

### 2.4. D—Increased Alveolar PO_2_

HFT can be used just as effectively without the addition of additional oxygen. However, oxygen therapy is one of the most commonly used drugs in hospitals and is highly used at home. More than 1.5 million people worldwide with a variety of respiratory disorders use long-term oxygen therapy to enhance their quality of life and prolong survival [49,50]. The difference between low- or moderate-flow devices, such as standard nasal cannulas or face masks, and HFT is that high flows maintain a stable (and high, when necessary) FiO_2_ by providing flow rates higher than spontaneous inspiratory flow (the patient’s ventilatory demand). This reduces the amount of entrained room air. As FiO_2_ is related to the proportion of pure oxygen coming from the interface (with 100% FiO_2_) and from the room air (21% FiO_2_), if the patient’s ventilatory demand exceeds the device’s flows, the patient will breathe some atmospheric air (entrainment effect) and FiO_2_ will decrease or become less accurate. Therefore, in low- to moderate-flow systems, usually called standard oxygen therapy (SOT), the “real” FiO_2_ depends on the patient’s breathing pattern and effort, determining the inspiratory flow [51]. Ritchie et al. demonstrated that when there is a large difference between the device flow rate and the patient’s peak inspiratory flow rate, the delivered FiO_2_ could decrease by 20% [52]. This advantage of HHFT could be relevant for a COPD patient undergoing home rehabilitation or exercise, or for patients during daily activities. During HFT, patients with COPD and exercise limitations are able to exercise longer with less dyspnea, a better breathing pattern and SaO_2_, less muscular fatigue and lower arterial pressure [53,54]. Comparable to COPD, exercise tolerance during HFT was investigated in stable patients with a prior diagnosis of idiopathic pulmonary fibrosis (IPF). Harada et al. enrolled 24 stable (no infection or acute exacerbation within 3 months) patients with exertional dyspnea (modified Medical Research Council scores (mMRC) 1–3) and exercise-induced oxygen desaturation (percutaneous oxygen saturation [SpO_2_] < 90% during a 6-min walking test, 6MWT). Following a “baseline” cycle ergometry test, all recruited patients underwent the same test randomly utilizing HFT and SOT (using a Venturi mask). Exercise duration, leg fatigue, and the lowest SpO_2_ attained throughout the test all improved during HFT as compared to SOT. There was no significant difference in dyspnea or heart rate between the two groups, and no adverse effects were reported [17].

## 3. Effects of HHFT on COPD Exacerbations and Economic Impact

COPD is a disease that has a significant socioeconomic impact. The high costs are primarily driven by the exacerbations that entail hospitalization and the use of primary care resources, which increase with the disease severity. Given the mechanisms discussed above, HFT may be useful in the home management of patients with COPD to reduce exacerbations [18,19]. The available evidence on the effects of HHFT on the exacerbation rate is limited. However, some findings may indicate that long-term domiciliary HFT treatment could be cost-effective [11,13,55]. Storgaard et al. investigated the long-term effects of HHFT in patients with COPD treated with LTOT [13]. Two hundred patients were randomized into usual care (only LTOT) or HHFT plus LTOT. The average daily use of HHFT plus LTOT was 6 h per day. HHFT plus LTOT treatment reduced the acute exacerbation rate (3.12 versus 4.95 patients per year, *p* = 0.001), hospital admissions (0.79 versus 1.39 patients per year for 12- versus 1-month use of HFT + LTOT, respectively; *p* = 0.001), and symptoms in patients with COPD with hypoxemic chronic failure. Nagata et al. conducted a multicenter crossover trial in 32 adults with stable hypercapnic COPD, comparing HHFT plus LTOT vs. LTOT only [11]. Participants were randomly assigned to either 6 weeks of HHFT plus LTOT or LTOT only, followed by 6 weeks of either LTOT only or HHFT plus LTOT. The primary goal of the study was to examine their quality of life; however, they also examined the exacerbations and economic benefit. Although the sample size was small and the observation time too short to adequately assess any benefit of HHFT on exacerbations, three participants who received LTOT only experienced acute exacerbations, while those treated with HHFT and LTOT did not experience any. More research is required to determine how HHFT affects prognosis and cost-effectiveness. Still, even a small benefit may result in a significant reduction in the economic and health burden.

## 4. HFT Role in the Management of Palliative Patients

A wide range of diseases require palliative care. The majority of adults who need palliative care have chronic illnesses such as cardiovascular disease, cancer, or chronic respiratory disease. Many other conditions, however, may necessitate palliative care. Providing early palliative care reduces hospitalizations and healthcare utilization. The general goals of palliative care are to relieve pain and other distressing symptoms, such as breathlessness, provide a supportive environment to assist patients in living as actively as possible until death, enhance quality of life, and possibly positively influence the course of the illness. Palliative care is frequently confused with hospice care, also known as end-of-life status. One of the primary distinctions between palliative care and end-of-life status is that in palliative care, treatment begins with the diagnosis of a terminal disease and with the goal of affirming life but accepting death as a natural process. Hospice care starts after disease treatment has ceased. Not all patients receiving palliative care are at the end-of-life or hospice stage. In the same manner, not all patients receiving palliative care have a do-not-intubate (DNI) or do-not-resuscitate order (DNR). Although some patients receiving palliative care may have a DNI directive, it is possible that other patients receiving palliative care do not have the same directive.

Pain and dyspnea are two of the symptoms most commonly faced by patients receiving palliative care. Although guidelines do not provide strong evidence to support the use of oxygen as a relief for non-hypoxemic patients [56,57], supplemental oxygen is widely prescribed in palliative care even when patients are not hypoxemic. Interestingly, it has been proposed that a relevant degree of comfort can be provided by placing a fan in front of a patient’s face [58]. Hiu et al. conducted a randomized clinical trial to assess the effect of flow rate (high vs. low) and gas (oxygen vs. air) on exertional dyspnea in non-hypoxemic cancer patients [20]. Forty-five non-hypoxemic patients with evidence of primary or secondary lung involvement and an average Modified Borg Dyspnea Scale (mMRC) of at least 4/10 were enrolled. High-flow oxygen (HFOx) and high-flow therapy (HFAir) were delivered through a nasal cannula, with flow gas titrated between 20 and 70 L/m according to patient tolerance. Low-flow oxygen (LFOx) and low-flow air (LFAir) were provided at 2 L/minute using a standard nasal cannula. Compared to LFAir, both HFOx and LFOx were able to decrease dyspnea; HFAir alone has no effect on dyspnea management compared to LFair. The LFOx group had improved exertional dyspnea compared with the LFAir group, suggesting that oxygenation, even at a low flow rate, may have a positive impact on exertional dyspnea even in the non-hypoxemic. The major questionable limitation of the study was that FiO_2_ was higher in the HFOx group than in the LFOx group (100% vs. 28% approximately).

NIV is frequently used in patients with DNR or DNI orders because it may alleviate dyspnea or hypercapnia, when present. Furthermore, NIV may be justified in selected patients when there is a reversible cause of respiratory failure and there is a chance of survival when the assessment of the reversibility of the respiratory failure is achieved [59]. Despite its benefits in terms of symptom relief and short-term survival in the acute setting [60], NIV may sometimes be harmful, due to the possibility of increasing suffering, discomfort or because it could fail to alleviate dyspnea, or when it interferes with communication with loved ones, particularly in some patients in “end of life” stages [61,62]. In these situations, NIV should be avoided. Instead, HFT, apart from providing steady FiO_2_, may be less claustrophobic, cause less skin deterioration, and not interfere with eating or talking. In palliative care, HFT has been shown to reduce dyspnea in hypoxemic patients with a DNI order in an end-of-life stage at the emergency department (ED) [63]. In 2018, Koyauchi et al. investigated the efficacy and tolerability of HFT and NIV in terminal patients with a DNI status and hypoxemic respiratory failure affected by interstitial lung fibrosis [64]. Fifty-four hospitalized patients received HFT and oxygen therapy for a median of 7 days at a median flow rate of 40 L/min, while 30 patients received NIV for a median time of 5 days. Patients treated with HFT had lower rates of temporary interruption and discontinuation. Similarly, compared to the NIV group, these patients were significantly better able to eat and communicate before death and experienced fewer adverse events. Although dyspnea scores did not differ significantly between groups, the respiratory rate decreased significantly following HFT. Nevertheless, there is currently no evidence that HFT is superior to NIV for palliative care. Indeed, there is little proof of HHFT in palliative care [65,66]. Further research is needed to identify its role, particularly at home. In addition, in some countries, there are restrictive regulations regarding HHTF prescription that deny access to this resource for palliative treatment.

## 5. Differences between Home and Hospital Implementation and Types of Home Devices

The primary distinction between HFT equipment used in hospitals and that used for home application is that home devices all use turbines to generate high flow, whereas the devices used in hospitals use various other methods (air–oxygen blender devices and entrainment systems, also called Venturi Systems) [67]. As a result, during HHFT, FiO_2_ is a “flow-dependent” parameter. As oxygen is supplied via a low-pressure system, any increase in flow will reduce FiO_2_, and vice versa (Table 2). High-flow therapy (HFT) is the administration of gas flows above 15 L/min. However, flow rates of 30–40 L/m are most commonly used during HHFT. However, differently from an acute setting, most of the physiological benefits of HFT are achieved with lower flow rates in stable COPD patients, as demonstrated by short-term physiological studies [11,45]. Pinkham and colleagues recently demonstrated in a bench model that HFT clearance of expired gas from nasal cavities occurs primarily at the end of expiration. Thus, in stable patients with a lower respiratory rate, efficient clearance can be accomplished using lower HFT rates that are also more comfortable [68]. When delivering HHFT via a nasal cannula, the size of the cannula should be chosen so that it does not occlude more than 50% of the nostril. The features offered by HHFT devices are similar. Table 3 summarizes the three most common HHTF devices. All are made up of a turbine unit with an integrated thermo-humidifier that, under ideal conditions, should deliver 33 mg/L absolute humidity and 100% relative humidity at 37 °C. The difference between them is based on their ability to deliver different maximum flow rates (e.g., up to 60 L/m or up to 40 L/m). They all have a display where it is possible to monitor the set data, and some give the possibility to monitor both FiO_2_ (usually in clinical mode) and saturation and heart rate (usually by connecting an external oximeter) as well as patient compliance (hours of use, therapy report). It is always recommended to read the clinic manuals for each device for further details.

Because active thermo-humidification is required, it is nearly impossible to achieve device autonomy through a battery. In addition, most devices use a pass-over system to achieve thermo-humidification, which consumes even more energy. However, some devices permit the connection of an external battery. Recently, a device with a 20-min autonomy was introduced to the market (currently for use in hospitals) (depending on the flow rate, temperature, and humidity).

## 6. Conclusions

As we have discussed, despite several potential positive physiological mechanisms, the use of HHFT in chronic hypercapnia needs still to be confirmed in larger randomized controlled trials.

Promising results were obtained when HHFT was compared with SOT, both in terms of reducing the level of PaCO_2_ and saving costs.

In the only direct comparison with NIV, HHFT resulted in similar clinical results, but it was not assessed whether there is or is not a “critical” PaCO_2_ threshold above or below which one of the two methods is preferable. Indeed, studies suggest that HHFT may be used during NIV intervals instead of SOT.

There could also be a rationale for using heated and humidified devices in those patients for whom dehydration may either trigger airway inflammation leading to emphysema or cause the accumulation of secretions in those with bronchiectasis.

HFT is a relatively new therapy that may have a place in palliative care. Despite the fact that to date there is little evidence to support its use in this scenario, it can be beneficial because it is well tolerated, even over long periods of time, with few adverse events that can be managed outside of the hospital.

## Figures and Tables

**Table 1:** HHFT studies cited in the text.

	Study Population	Study Design	HFT Settings	Outcomes	Results	Hypothesized Physiologic Effect
**Rea et al. (2010)** **[9]**	108 stable patients diagnosed with COPD or bronchiectasis.	Randomized open-label controlled trial: HFT vs. usual care.	Flow: 20–25 L/m. HFT use: 1.6 ± 0.67 h/d. Temperature: 37 °C.	Exacerbation rate, time to first exacerbation, number of exacerbated days and hospital admissions, change in QoL scores, lung function, 6MWT, and inflammatory markers (sputum cell counts) over 12 months.	HFT reduced exacerbation rate, increased time to first exacerbation, reduced exacerbation frequency, improved QoL scores and lung function.	Improvement in mucociliary clearance. Washout of dead space. PEEP effect.
**Hasani et al. (2008)** **[10]**	10 patients diagnosed with idiopathic bronchiectasis in stable phase.	Physiologic study: HFT in bronchiectasis patients.	Flow: 20–25 L/m. HFT use: >3 h/d. Temperature: 37 °C.	Clearance of radioactively tagged 99mTc-polystyrene aerosolized particles.	HFT improved lung mucociliary clearance.	Improvement in mucociliary clearance.
**Nagata et al. (2018)** **[11]**	32 patients diagnosed with stable hypercapnic COPD.	Multicenter, randomized crossover trial: HFT + LTOT vs. LTOT (6 weeks for each trial).	Flow: 29.2 ± 1.9 L/min (group A); 30.3 ± 4.6 L/min (group B). HFT use: 7.1 ± 1.5 h/d (group A); 8.6 ± 2.9 h/d (group B).	Variations in QoL scores, dyspnea scores, ABG, nocturnal PtcCO_2_, SpO_2_, PFTs, 6MWT, and physical activity. AECOPD.	HFT improved QoL scores, PaCO_2_, pH, and nocturnal PtcCO_2_.	Dead space washout.
**Fraser et al. (2016)** **[12]**	30 stable COPD patients in LTOT	Randomized physiologic crossover trial: HFT vs. LTOT (20 min for each period).	Flow: 30 L/m.	Variations in TcO_2_, TcCO_2_, SpO_2_, Vt, MV, RR, I:E ratio, EELI, HR, dyspnea, and comfort.	HFT reduced TcO_2_, TcCO_2_, RR, and I:E ratio. HFT increased Vt and EELI.	Dead space washout, PEEP effect.
**Storgaard et al. (2018)** **[13]**	200 COPD patients with chronic hypoxemic respiratory failure.	Randomized clinical trial: HFT + LTOT vs. LTOT.	Flow: 20 L/m. HFT use: 6h/d. Temperature: not available.	Rate of AECOPD, hospital admissions, variations in dyspnea, QoL scores, PaCO_2_, all-cause mortality and exercise performance at 12 months.	HFT reduced AECOPD rate, improved dyspnea, QoL scores, and 6MWT distance. HFT decreased PaCO_2_ at 12 months.	Improvement in mucociliary clearance.
**Storgaard et al. (2020)** **[14]**	74 COPD patients with persistent hypercapnic failure.	Post-hoc analysis of Ref [13]: HFT + LTOT vs. LTOT.	Flow: 20 L/m. HFT use: 6.9 h/d. Temperature: not available.		PaCO_2_ decreased in HFT + LTOT group while it increased in LTOT group.	Clearance of CO_2_ from the anatomical dead space.
**Pisani et al. (2020)** **[15]**	50 COPD or COPD/OSA hypercapnic patients recovered from an acute exacerbation.	Interventional study: HFT in persistent hypercapnia following acute exacerbation.	Flow: 33.5 ± 3.2 L/min. HFT use: 8h/d + night-time.	Variations in ABG, RR.	HFT reduced RR. HFT reduced pCO_2_ only in pure COPD patients.	Dead space washout.
**Bräunlich et al. (2019)** **[16]**	102 COPD patients with stable daytime hypercapnia.	Multi-centered, randomized controlled crossover trial: HFT vs. NIV (6 weeks for each trial).	Flow: 19.8 ± 0.6 L/min.	Variations in pCO_2_, lung function, QoL scores, 6MWT, and duration of device use.	HFT was effective as NIV in terms of pCO_2_ reduction (slight tendency in favor of NIV) and QoL scores.	Dead space washout.
**Harada et al. (2022)** **[17]**	24 patients diagnosed with IPF and exercise-induced oxygen desaturation.	Prospective, randomized crossover trial: HFT vs. SOT (Venturi mask).	Flow: 60 L/m. FiO2: 50%. Temperature: 37 °C.	Endurance time, SpO_2_, dyspnea leg fatigue, HR, comfort.	HFT improved exercise duration, leg fatigue, and minimum SpO_2_.	Ensured adequate inspiratory flow.
**Nagata et al. (2022) [18]**	104 patients diagnosed with COPD (GOLD 2-4).	Randomized clinical trial: HFT + LTOT vs. LTOT.	Flow: 28.5 ± 4.57 L/min. HFT use: 7.3 ± 3.0 h/d. Temperature: 37 °C; modified according to patient’s comfort.	Moderate/severe AECOPD rate. Variations in ABG, pulmonary function, QoL scores.	HFT reduced the rate of moderate/severe AECOPD, improved time to first exacerbation, QoL scores, and pulmonary function.	Improvement in secretory clearance. Improve muscle weakness.
**Good et al. (2020)** **[19]**	45 patients diagnosed with bronchiectasis.	Post-hoc analysis of Ref [9]: HFT vs. usual care.	Flow: 20–25 L/m. HFT use: 1.7 h/d. Temperature: 37 °C.		HFT reduced exacerbation rate and improved QoL scores.	Improvement in mucociliary clearance.
**Hui et al. (2020)** **[20]**	44 non-hypoxemic patients diagnosed with cancer involving the lung.	Double-blind, randomized clinical trial: HFox vs. HFair vs. LFox vs. LFair.	HFox Flow: 48 ± 12 L/m. HFair flow: 46 ± 11 L/minute. Temperature: 35 °C.	Dyspnea, exercise duration, leg discomfort during exercise test.	HFox and LFox reduced exertional dyspnea compared to LFair. HFox improved exercise capacity compared to LFair.	

COPD: chronic obstructive pulmonary disease; HFT: high-flow therapy; LTOT: long-term oxygen therapy; AECOPD: acute exacerbation of chronic obstructive pulmonary disease; ABG: arterial blood gas; QoL: quality of life; 6MWT: six-minute walking test; HFAir: high-flow air; HFOx: high-flow oxygen; LFAir: low-flow air; LFOx: low-flow oxygen; HR: heart rate; RR: respiratory rate; OSA: obstructive sleep apnoea; TcO_2_: transcutaneous oxygen; TcCO_2_: transcutaneous carbon dioxide; SpO_2_: Percutaneous oxygen saturation; Vt: tidal volume; MV: minute volume; I:E ratio; EELI: end-expiratory lung impedance.

**Table 2:** Estimated FiO_2_ according to domiciliary flow setting.

FiO_2_ *						Flow (L/m)					
		15	20	25	30	35	40	45	50	55	60
**Concentrator oxygen flow rate in L/m**	**1**	**26**	**25**	**24**	**24**	**23**	**23**	**23**	**23**	**22**	**22**
	**2**	**32**	**29**	**27**	**26**	**26**	**25**	**25**	**24**	**24**	**24**
	**3**	**37**	**33**	**31**	**29**	**28**	**27**	**26**	**26**	**25**	**25**
	**4**	**42**	**37**	**34**	**32**	**30**	**29**	**28**	**27**	**27**	**26**
	**5**	**47**	**41**	**37**	**34**	**32**	**31**	**30**	**29**	**28**	**28**
	**6**	**53**	**45**	**40**	**37**	**35**	**33**	**32**	**31**	**30**	**29**
	**7**	**58**	**49**	**43**	**40**	**37**	**35**	**33**	**32**	**31**	**30**
	**8**	**63**	**53**	**46**	**42**	**39**	**37**	**35**	**34**	**33**	**32**
	**9**	**68**	**56**	**50**	**45**	**42**	**39**	**37**	**35**	**34**	**33**
	**10**	**73**	**60**	**53**	**47**	**44**	**41**	**39**	**37**	**36**	**34**
	**11**	**78**	**64**	**56**	**50**	**46**	**43**	**41**	**39**	**37**	**36**
	**12**	**82**	**68**	**59**	**53**	**48**	**45**	**42**	**40**	**38**	**37**
	**13**	**87**	**71**	**62**	**55**	**50**	**47**	**44**	**42**	**40**	**38**
	**14**	**92**	**75**	**65**	**58**	**53**	**49**	**46**	**43**	**41**	**40**
	**15**	**93**	**79**	**68**	**60**	**55**	**51**	**47**	**45**	**43**	**41**

(Note: It is always recommended to monitor the efficiency of oxygen therapy (i.e., in terms of SaO_2_) and not prescribe without particular control). * Actual data calculated using a medical oxygen cylinder with a purity of 99.5%. FiO_2_ may vary ±1–2% regarding the devices.

**Table 3:** Main characteristics of HHFT devices.

	MyAirvo™ (Fisher and Paykel)	Lumis™ HFT (ResMed)	H-FLOW™ (Medical Products Research S.r.l.)
**Flow range**	10–60 L/min.	15–40 L/min.	10–60 L/min.
**Concentrator oxygen flow rate**	Up to 15 L/m.	Up to 15 L/m.	Up 15 L/m.
**Humidification and** **temperature settings**	Pass-over system. Reusable or auto-fill water chamber. Heated breathing tube T° target setting = 31 °C, 34 °C, 37 °C. Humidity performance of 33 mg/L at 37 °C, and of 12 mg/L at 31 °C and 34 °C.	Pass-over system. Reusable water chamber. Heated breathing tube (ClimateLineAir™ + tube cover) T° target setting of 31 °C, 34 °C, 37 °C). Humidity level from 1 to 5 (from the lowest to the highest humidity).	Pass-over system. Reusable or auto-fill water chamber. Heated breathing tube T° target setting = 31 °C, 34 °C, 37 °C. Humidity performance of 33 mg/L at 37 °C, and of 12 mg/L at 31 °C and 34 °C.
**Interface**	Optiflow +™ nasal cannula (3 sizes). Optiflow Tracheostomy™ interface.	AcuCare™ nasal cannula or generic high-flow nasal cannula (note: tracheostomy use is contraindicated for Lumis HFT).	Generic high-flow nasal cannula. Generic high-flow tracheostomy interface.
**Alarms**	Yes.	Messages and warnings.	Yes.
**Patient compliance monitoring**	USB port for data download therapy, Infosmart™.	SD card for data download therapy (ResScan™ and Airview™- telemonitoring). Soon, the ability to remotely edit settings will be available.	SD card.
**Possibility of connecting a built-in oximeter**	No.	Yes, Air10 oximeter adapter (Nonin-XPod^®^, Global Headquarters, Plymouth, UK).	Yes.
**Weight**	2.2 kg.	1.29 kg.	3 kg.

T°: Temperature; °C: Celsius degrees; kg: kilograms.

## Data Availability

Not applicable.

## References

[1] Chikata Y., Izawa M., Okuda N., Itagaki T., Nakataki E., Onodera M., Imanaka H., Nishimura M. (2014). Humidification performance of two high-flow nasal cannula devices: A bench study. Respir. Care.

[2] Delorme M., Bouchard P.-A., Simard S., Lellouche F. (2021). Hygrometric Performances of Different High-Flow Nasal Cannula Devices: Bench Evaluation and Clinical Tolerance. Respir. Care.

[3] Locke R.G., Wolfson M.R., Shaffer T.H., Rubenstein S.D., Greenspan J.S. (1993). Inadvertent administration of positive end-distending pressure during nasal cannula flow. Pediatrics.

[4] Sreenan C., Lemke R.P., Hudson-Mason A., Osiovich H. (2001). High-flow nasal cannulae in the management of apnea of prematurity: A comparison with conventional nasal continuous positive airway pressure. Pediatrics.

[5] Dewan N.A., Bell C.W. (1994). Effect of low flow and high flow oxygen delivery on exercise tolerance and sensation of dyspnea. A study comparing the transtracheal catheter and nasal prongs. Chest.

[6] Kernick J., Magarey J. (2010). What is the evidence for the use of high flow nasal cannula oxygen in adult patients admitted to critical care units? A systematic review. Aust. Crit. Care.

[7] Rochwerg B., Granton D., Wang D.X., Helviz Y., Einav S., Frat J.P., Mekontso-Dessap A., Schreiber A., Azoulay E., Mercat A. (2019). High flow nasal cannula compared with conventional oxygen therapy for acute hypoxemic respiratory failure: A systematic review and meta-analysis. Intensiv. Care Med..

[8] Fernando S.M., Tran A., Sadeghirad B., Burns K.E.A., Fan E., Brodie D., Munshi L., Goligher E.C., Cook D.J., Fowler R.A. (2021). Noninvasive respiratory support following extubation in critically ill adults: A systematic review and network meta-analysis. Intensiv. Care Med..

[9] Rea H., McAuley S., Jayaram L., Garrett J., Hockey H., Storey L., O’Donnell G., Haru L., Payton M., O’Donnell K. (2010). The clinical utility of long-term humidification therapy in chronic airway disease. Respir. Med..

[10] Hasani A., Chapman T., McCool D., Smith R., Dilworth J., Agnew J. (2008). Domiciliary humidification improves lung mucociliary clearance in patients with bronchiectasis. Chronic Respir. Dis..

[11] Nagata K., Kikuchi T., Horie T., Shiraki A., Kitajima T., Kadowaki T., Tokioka F., Chohnabayashi N., Watanabe A., Sato S. (2018). Domiciliary High-Flow Nasal Cannula Oxygen Therapy for Patients with Stable Hypercapnic Chronic Obstructive Pulmonary Disease. A Multicenter Randomized Crossover Trial. Ann. Am. Thorac. Soc..

[12] Fraser J.F., Spooner A.J., Dunster K.R., Anstey C., Corley A. (2016). Nasal high flow oxygen therapy in patients with COPD reduces respiratory rate and tissue carbon dioxide while increasing tidal and end-expiratory lung volumes: A randomised crossover trial. Thorax.

[13] Storgaard L.H., Hockey H.-U., Laursen B.S., Weinreich U.M. (2018). Long-term effects of oxygen-enriched high-flow nasal cannula treatment in COPD patients with chronic hypoxemic respiratory failure. Int. J. Chronic Obstr. Pulm. Dis..

[14] Storgaard L.H., Hockey H.-U., Weinreich U.M. (2020). Development in PaCO_2_ over 12 months in patients with COPD with persistent hypercapnic respiratory failure treated with high-flow nasal cannula—Post-hoc analysis from a randomised controlled trial. BMJ Open Respir. Res..

[15] Pisani L., Betti S., Biglia C., Fasano L., Catalanotti V., Prediletto I., Comellini V., Bacchi-Reggiani L., Fers S.N. (2020). Effects of high-flow nasal cannula in patients with persistent hypercapnia after an acute COPD exacerbation: A prospective pilot study. BMC Pulm. Med..

[16] Bräunlich J., Dellweg D., Bastian A., Budweiser S., Randerath W., Triché D., Bachmann M., Kähler C., Bayarassou A.H., Mäder I. (2019). Nasal high-flow versus noninvasive ventilation in patients with chronic hypercapnic COPD. Int. J. Chronic Obstr. Pulm. Dis..

[17] Harada J., Nagata K., Morimoto T., Iwata K., Matsunashi A., Sato Y., Tachikawa R., Ishikawa A., Tomii K. (2021). Effect of high-flow nasal cannula oxygen therapy on exercise tolerance in patients with idiopathic pulmonary fibrosis: A randomized crossover trial. Respirology.

[18] Nagata K., Horie T., Chohnabayashi N., Jinta T., Tsugitomi R., Shiraki A., Tokioka F., Kadowaki T., Watanabe A., Fukui M. (2022). Home High-Flow Nasal Cannula Oxygen Therapy for Stable Hypercapnic COPD: A Randomized Clinical Trial. Am. J. Respir. Crit. Care Med..

[19] Good W.R., Garrett J., Hockey H.U., Jayaram L., Wong C., Rea H. (2021). The role of high-flow nasal therapy in bronchiectasis: A *post hoc* analysis. ERJ Open Res..

[20] Hui D., Mahler D.A., Larsson L., Wu J., Thomas S., Harrison C.A., Hess K., Lopez-Mattei J., Thompson K., Gomez D. (2020). High-Flow Nasal Cannula Therapy for Exertional Dyspnea in Patients with Cancer: A Pilot Randomized Clinical Trial. Oncol..

[21] Goligher E.C., Slutsky A.S. (2017). Not Just Oxygen? Mechanisms of Benefit from High-Flow Nasal Cannula in Hypoxemic Respiratory Failure. Am. J. Respir. Crit. Care Med..

[22] Pisani L., Vega M.L. (2017). Use of Nasal High Flow in Stable COPD: Rationale and Physiology. COPD J. Chronic Obstr. Pulm. Dis..

[23] Simel D.L., Mastin J.P., Pratt P.C., Wisseman C.L., Shelburne J.D., Spock A., Ingram P. (1984). Scanning electron microscopic study of the airways in normal children and in patients with cystic fibrosis and other lung diseases. Pediatr. Pathol..

[24] Salah B., Dinh-Xuan A.T., Fouilladieu J.L., Lockhart A., Regnard J. (1988). Nasal mucociliary transport in healthy subjects is slower when breathing dry air. Eur. Respir. J..

[25] Mall M.A., Harkema J.R., Trojanek J.B., Treis D., Livraghi A., Schubert S., Zhou Z., Kreda S.M., Tilley S.L., Hudson E.J. (2008). Development of Chronic Bronchitis and Emphysema in β-Epithelial Na^+^ Channel–Overexpressing Mice. Am. J. Respir. Crit. Care Med..

[26] Cuquemelle E., Pham T., Papon J.-F., Louis B., Danin P.-E., Brochard L. (2012). Heated and humidified high-flow oxygen therapy reduces discomfort during hypoxemic respiratory failure. Respir. Care.

[27] Chanques G., Constantin J.-M., Sauter M., Jung B., Sebbane M., Verzilli D., Lefrant J.-Y., Jaber S. (2009). Discomfort associated with underhumidified high-flow oxygen therapy in critically ill patients. Intensiv. Care Med..

[28] Fontanari P., Burnet H., Zattara-Hartmann M.C., Jammes Y. (1996). Changes in airway resistance induced by nasal inhalation of cold dry, dry, or moist air in normal individuals. J. Appl. Physiol..

[29] Fontanari P., Zattara-Hartmann M., Burnet H., Jammes Y. (1997). Nasal eupnoeic inhalation of cold, dry air increases airway resistance in asthmatic patients. Eur. Respir. J..

[30] Shepard J.W., Burger C.D. (1990). Nasal and oral flow-volume loops in normal subjects and patients with obstructive sleep apnea. Am. Rev. Respir. Dis..

[31] On L.S., Boonyongsunchai P., Webb S., Davies L., Calverley P.M.A., Costello R.W. (2001). Function of pulmonary neuronal m_2_ muscarinic receptors in stable chronic obstructive pulmonary disease. Am. J. Respir. Crit. Care Med..

[32] Polverino E., Goeminne P.C., McDonnell M.J., Aliberti S., Marshall S.E., Loebinger M.R., Murris M., Cantón R., Torres A., Dimakou K. (2017). European Respiratory Society guidelines for the management of adult bronchiectasis. Eur. Respir. J. Off. J. Eur. Soc. Clin. Respir. Physiol..

[33] Crimi C., Noto A., Cortegiani A., Campisi R., Heffler E., Gregoretti C., Crimi N. (2020). High Flow Nasal Therapy Use in Patients with Acute Exacerbation of COPD and Bronchiectasis: A Feasibility Study. COPD J. Chronic Obstr. Pulm. Dis..

[34] Birk R., Händel A., Wenzel A., Kramer B., Aderhold C., Hörmann K., Stuck B.A., Sommer J.U. (2017). Heated air humidification versus cold air nebulization in newly tracheostomized patients. Head Neck.

[35] Dolidon S., Dupuis J., Valencia L.-C.M., Salaün M., Thiberville L., Muir J.-F., Cuvelier A., Patout M. (2019). Characteristics and outcome of patients set up on high-flow oxygen therapy at home. Ther. Adv. Respir. Dis..

[36] Möller W., Celik G., Feng S., Bartenstein P., Meyer G., Eickelberg O., Schmid O., Tatkov S. (2015). Nasal high flow clears anatomical dead space in upper airway models. J. Appl. Physiol..

[37] Möller W., Feng S., Domanski U., Franke K.-J., Celik G., Bartenstein P., Becker S., Meyer G., Schmid O., Eickelberg O. (2017). Nasal high flow reduces dead space. J. Appl. Physiol..

[38] Delorme M., Bouchard P.-A., Simon M., Simard S., Lellouche F. (2020). Physiologic Effects of High-Flow Nasal Cannula in Healthy Subjects. Respir. Care.

[39] Spoletini G., Mega C., Pisani L., Alotaibi M., Khoja A., Price L.L., Blasi F., Nava S., Hill N.S. (2018). High-flow nasal therapy vs standard oxygen during breaks off noninvasive ventilation for acute respiratory failure: A pilot randomized controlled trial. J. Crit. Care.

[40] Groves N., Tobin A. (2007). High flow nasal oxygen generates positive airway pressure in adult volunteers. Aust. Crit. Care.

[41] Parke R.L., Bloch A., McGuinness S.P. (2015). Effect of Very-High-Flow Nasal Therapy on Airway Pressure and End-Expiratory Lung Impedance in Healthy Volunteers. Respir. Care.

[42] Parke R.L., McGuinness S.P. (2013). Pressures delivered by nasal high flow oxygen during all phases of the respiratory cycle. Respir. Care.

[43] Mündel T., Feng S., Tatkov S., Schneider H. (2013). Mechanisms of nasal high flow on ventilation during wakefulness and sleep. J. Appl. Physiol..

[44] Vieira F., Bezerra F.S., Coudroy R., Schreiber A., Telias I., Dubo S., Cavalot G., Pereira S.M., Piraino T., Brochard L.J. (2022). High-flow nasal cannula compared with continuous positive airway pressure: A bench and physiological study. J. Appl. Physiol..

[45] Pisani L., Fasano L., Corcione N., Comellini V., Musti M.A., Brandao M., Bottone D., Calderini E., Navalesi P., Nava S. (2017). Change in pulmonary mechanics and the effect on breathing pattern of high flow oxygen therapy in stable hypercapnic COPD. Thorax.

[46] Yang Y., Wei L., Wang S., Ke L., Zhao H., Mao J., Li J., Mao Z. (2020). The effects of pursed lip breathing combined with diaphragmatic breathing on pulmonary function and exercise capacity in patients with COPD: A systematic review and meta-analysis. Physiother. Theory Pract..

[47] Thomas M., Joshi R., Cave G. (2021). How Much PEEP Does High Flow Deliver via Tracheostomy? A Literature Review and Benchtop Experiment. Crit. Care Res. Pract..

[48] Natalini D., Grieco D.L., Santantonio M.T., Mincione L., Toni F., Anzellotti G.M., Eleuteri D., Di Giannatale P., Antonelli M., Maggiore S.M. (2019). Physiological effects of high-flow oxygen in tracheostomized patients. Ann. Intensiv. Care.

[49] Jacobs S.S., Lederer D.J., Garvey C.M., Hernandez C., Lindell K.O., McLaughlin S., Schneidman A.M., Casaburi R., Chang V., Cosgrove G.P. (2018). Optimizing Home Oxygen Therapy. An Official American Thoracic Society Workshop Report. Ann. Am. Thorac. Soc..

[50] Hardinge M., Annandale J., Bourne S., Cooper B., Evans A., Freeman D., Green A., Hippolyte S., Knowles V., MacNee W. (2015). British Thoracic Society guidelines for home oxygen use in adults. Thorax.

[51] Wagstaff T.A.J., Soni N. (2007). Performance of six types of oxygen delivery devices at varying respiratory rates. Anaesthesia.

[52] Ritchie J.E., Williams A.B., Gerard C., Hockey H. (2011). Evaluation of a humidified nasal high-flow oxygen system, using oxygraphy, capnography and measurement of upper airway pressures. Anaesth. Intensiv. Care.

[53] Chatila W., Nugent T., Vance G., Gaughan J., Criner G.J. (2004). The effects of high-flow vs low-flow oxygen on exercise in advanced obstructive airways disease. Chest.

[54] Cirio S., Piran M., Vitacca M., Piaggi G., Ceriana P., Prazzoli M., Carlucci A. (2016). Effects of heated and humidified high flow gases during high-intensity constant-load exercise on severe COPD patients with ventilatory limitations. Respir Med..

[55] Sorensen S.S., Storgaard L.H., Weinreich U.M. (2021). Cost-Effectiveness of Domiciliary High Flow Nasal Cannula Treatment in COPD Patients with Chronic Respiratory Failure. Clin. Outcomes Res..

[56] Lanken P.N., Terry P.B., DeLisser H.M., Fahy B.F., Hansen-Flaschen J., Heffner J.E., Levy M., Mularski R.A., Osborne M.L., Prendergast T.J. (2008). An official american thoracic society clinical policy statement: Palliative care for patients with respiratory diseases and critical illnesses. Am. J. Respir. Crit. Care Med..

[57] Hardinge M., Suntharalingam J., Wilkinson T., British Thoracic Society (2015). Guideline update: The British Thoracic Society Guidelines on home oxygen use in adults. Thorax.

[58] Galbraith S., Fagan P., Perkins P., Lynch A., Booth S. (2010). Does the use of a handheld fan improve chronic dyspnea? A randomized, controlled, crossover trial. J. Pain Symptom Manag..

[59] American Thoracic Society, European Respiratory Society, European Society of Intensive Care Medicine, Société de Réanimation de Langue Française (2001). International Consensus Conferences in Intensive Care Medicine: Noninvasive positive pressure ventilation in acute respiratory failure. Am. J. Respir Crit Care Med..

[60] Meduri G.U., Fox R.C., Abou-Shala N., Leeper K.V., Wunderink R.G. (1994). Noninvasive mechanical ventilation via face mask in patients with acute respiratory failure who refused endotracheal intubation. Crit. Care Med..

[61] Curtis J.R., Cook D.J., Sinuff T., White D.B., Hill N., Keenan S.P., Benditt J.O., Kacmarek R., Kirchhoff K.T., Levy M.M. (2007). Noninvasive positive pressure ventilation in critical and palliative care settings: Understanding the goals of therapy. Crit. Care Med..

[62] Rochwerg B., Brochard L., Elliott M.W., Hess D., Hill N.S., Nava S., Navalesi P., Antonelli M., Brozek J., Conti G. (2017). Official ERS/ATS clinical practice guidelines: Noninvasive ventilation for acute respiratory failure. Eur. Respir. J..

[63] Ruangsomboon O., Dorongthom T., Chakorn T., Monsomboon A., Praphruetkit N., Limsuwat C., Surabenjawong U., Riyapan S., Nakornchai T., Chaisirin W. (2020). High-Flow Nasal Cannula Versus Conventional Oxygen Therapy in Relieving Dyspnea in Emergency Palliative Patients with Do-Not-Intubate Status: A Randomized Crossover Study. Ann. Emerg. Med..

[64] Koyauchi T., Hasegawa H., Kanata K., Kakutani T., Amano Y., Ozawa Y., Matsui T., Yokomura K., Suda T. (2018). Efficacy and Tolerability of High-Flow Nasal Cannula Oxygen Therapy for Hypoxemic Respiratory Failure in Patients with Interstitial Lung Disease with Do-Not-Intubate Orders: A Retrospective Single-Center Study. Respiration.

[65] Bode S., Grove G. (2020). Use of Humidified High Flow Nasal Oxygen in Community Palliative Care: A Case Report. Palliat. Med. Rep..

[66] Goda K., Kenzaka T., Kuriyama K., Hoshijima M., Akita H. (2020). End-of-life home care of an interstitial pneumonia patient supported by high-flow nasal cannula therapy: A case report. World J. Clin. Cases.

[67] Vega M., Pisani L. (2021). Nasal high flow oxygen in acute respiratory failure. Pulmonology.

[68] Pinkham M.I., Domanski U., Franke K.-J., Hartmann J., Schroeder M., Williams T., Nilius G., Tatkov S. (2022). Effect of respiratory rate and size of cannula on pressure and dead-space clearance during nasal high flow in patients with COPD and acute respiratory failure. J. Appl. Physiol..

